# Casting Light on Early Heart Failure: Unveiling the Prognostic Potential of the E/(e′ × s′) Index

**DOI:** 10.3390/diagnostics14040409

**Published:** 2024-02-13

**Authors:** Ioana Ionac, Mihai Andrei Lazar, Teodora Hoinoiu, Simina Crisan, Silvius Alexandru Pescariu, Ciprian Nicusor Dima, Constantin Tudor Luca, Cristian Mornos

**Affiliations:** 1 Doctoral School Medicine-Pharmacy, “Victor Babes” University of Medicine and Pharmacy, 2 Eftimie Murgu Sq., 300041 Timisoara, Romania; ionac.ioana@umft.ro (I.I.); urseanusimina@yahoo.com (S.C.); constantin.luca@umft.ro (C.T.L.); mornos.cristian@umft.ro (C.M.); 2 Department VI Cardiology—Cardiology Clinic, Institute for Cardiovascular Diseases of Timișoara, “Victor Babes” University of Medicine and Pharmacy, 2 Eftimie Murgu Sq., 300041 Timisoara, Romania; lazar.mihai@umft.ro; 3 Department V, 1st Internal Medicine, Discipline of Clinical Practical Skills, “Victor Babes” University of Medicine and Pharmacy, No. 2 Eftimie Murgu Square, 300041 Timisoara, Romania; 4 Advanced Cardiology and Hemostaseology Research Center, “Victor Babes” University of Medicine and Pharmacy, No. 2 Eftimie Murgu Square, 300041 Timisoara, Romania; 5 Department VI Cardiology—Cardiovascular Surgery Clinic, Institute for Cardiovascular Diseases of Timișoara, “Victor Babeș” University of Medicine and Pharmacy from Timișoara, No. 2 Eftimie Murgu Square, 300041 Timișoara, Romania; alex.pescariu@yahoo.com (S.A.P.); dima.ciprian@umft.ro (C.N.D.); 6 Research Center of the Institute of Cardiovascular Diseases Timișoara, “Victor Babeș” University of Medicine and Pharmacy from Timișoara, No. 2 Eftimie Murgu Square, 300041 Timișoara, Romania

**Keywords:** tissue Doppler imaging, prognosis, early-stage heart failure, cardiac death, rehospitalization

## Abstract

It has been shown that patients with NYHA class I and II have a high morbidity and mortality burden. We investigated the value of a new tissue Doppler index, E/(e′ × s′), to predict cardiac events in the long-term follow-up of patients at an early stage of heart failure (HF). Sequential echocardiography was conducted on a consecutive cohort of 212 hospitalized HF patients, pre-discharged and with three-month follow-up. The primary end point consisted of cardiac death or readmission due to HF worsening. During follow-up, cardiac events occurred in 99 patients (46.7%). The first cardiac event was represented by cardiac death in 8 patients (3.8%) and readmission for HF in 91 patients (42.9%). A Kaplan–Meier analysis did not show a significantly different event-free survival rate between patients with NYHA class I and II. The composite end point was significantly higher in patients with an E/(e′ × s′) >1.6. The E/(e′ × s′) at discharge was the best independent predictor of cardiac events. Those exhibiting an E/(e′ × s′) > 1.6 at discharge, with a subsequent deterioration after three months, displayed the poorest prognosis concerning cardiac events, HF-related rehospitalization, and cardiac mortality (all *p* < 0.05). In early-stage HF patients, an E/(e′ × s′) > 1.6 emerged as a robust predictor of clinical outcomes, especially when coupled with a deterioration in condition.

## 1. Introduction

Heart failure (HF) is a chronic disease with a similar, if not worse, prognosis than other serious and life-threatening conditions, such as chronic kidney disease or cancer [1,2]. Despite the development of novel drugs and devices responsible for significant improvements in patient survival, the mortality rate remains high, with one in three patients dying within 1 year of hospitalization for HF and 40–50% within 5 years of diagnosis [1,2,3]. The poor outcome associated with left ventricular (LV) dysfunction results in the need to obtain prognostic information as soon as possible.

In daily practice, patients with HF are divided into functional classes based on the New York Heart Association (NYHA) classification. This symptomatic classification has been a major entry criterion for the clinical trials that support current HF treatment guidelines. It has been shown that patients with HF have increased mortality rates, irrespective of symptomatology and LV ejection fraction (EF). Providers may falsely believe that patients with milder symptoms have low morbidity and mortality, and those patients with advanced disease may be beyond help [3]. Patients with HF with preserved LVEFs and signs of an elevated left atrial (LA) pressure on echocardiography potentially have an increased risk of death or HF rehospitalization [4,5]. Even patients at asymptomatic early stages of HF show evidence of ongoing adaptive and maladaptive pathways [1,6]. Accordingly, patients with NYHA class I or II still have a relatively high morbidity and mortality burden. In a sub-analysis of the Digitalis Investigational Group trial, when 1863 subjects with NYHA I and II were matched to the same number of subjects with NYHA III and IV, the mortality rates were 34% versus 42%, and all-cause hospitalizations were 66% versus 71%, respectively [7]. Patients at an early stage of HF are ideal targets for complication prevention.

The entire echocardiographic evaluation of a patient referred with suspected HF must include a thorough assessment of LV function. All modalities, including tissue Doppler imaging (TDI) and conventional echocardiography, offer diagnostic hints [8]. As the most accurate echocardiographic predictor in an LV filling pressure assessment in various clinical contexts, the ratio of the early diastolic velocity of mitral annular motion (e′) to the early diastolic velocity of mitral inflow (E) has been proposed [9,10]. This ratio shows a continuous progression with increasing mean pulmonary capillary wedge pressure.

The analysis of the LV long-axis function demonstrates valuable additive information for a noninvasive assessment of HF prognosis [11]. Several years ago, our group proposed a new TDI index, E/(e′ × s′), that associates a marker of diastolic function (E/e′) and a parameter that explores LV systolic performance (systolic mitral annular velocity, s′) to assess the LV filling pressure [12]. We identified that a value > 1.6 for E/(e′ × s′) can be a good predictor of clinical outcome in patients with HF [13].

Our purpose has been to investigate the value of the E/(e′ × s′) ratio in predicting cardiac events in the long-term follow-up of patients at an early stage of HF (NYHA class I and II).

## 2. Materials and Methods

### 2.1. Study Population

#### Patients

We prospectively analyzed 500 consecutive patients with HF, in sinus rhythm, hospitalized in our clinic. Only patients with NYHA functional class I or II were selected for this study. Exclusion criteria were represented by inadequate echocardiographic images, symptoms or clinical and instrumental signs of acute coronary syndrome or myocarditis, coronary revascularization during follow-up, significant primary valvular heart disease (except mild forms not hemodynamically relevant), cardiac pacemaker/defibrillator, congenital heart disease, renal failure (serum creatinine > 1.3 mg/dL), malignant neoplasia, anemia (hemoglobin < 12 mg/dL in women and <13 mg/dL in men), and endocrine disorders (hypo- and hyperthyroidism, hyperaldosteronism). Pericardial disease, primary pulmonary hypertension, and aortic diseases were excluded based on echocardiography. The remaining 212 patients formed our study group. The study complied with the Declaration of Helsinki and was approved by the local research ethics committee. Patient group criteria are detailed in Table 1.

### 2.2. Echocardiography

Using Milwaukee, WI-based Vivid 9 General Electric equipment, echocardiography was performed within 24 h of hospital release after appropriate medical care. During the apical four-chamber and apical two-chamber views during ventricular end-systole (maximum LA size), the biplane area-length method was used to compute the LA volume, which was then indexed for body surface area. With the modified Simpson’s rule, LVEF was computed from apical two- and four-chamber images [14]. The mitral regurgitation’s regurgitant orifice area (ROA) and regurgitant volume (RV) were estimated [15]. With an apical four-chamber window and a 5 mm pulsed-sample Doppler volume placed in between the mitral valve tips, the transmitral flow patterns were recorded. During end-expiratory apnea, the E and late transmitral flow (A) were measured throughout the course of five cardiac cycles, and the average values were noted [10]. The global myocardial index (GMI) was calculated by dividing the ejection time by the sum of the isovolumic contraction and relaxation times [16]. In using continuous Doppler to measure the maximal regurgitant velocity at the tricuspid valve, the systolic pulmonary artery pressure (SPAP) was determined.

The TDI program was run in pulsed-wave Doppler mode, putting a 5 mm sample volume sequentially at the mitral annulus’ lateral and septal corners inside the apical four-chamber view [10]. During end-expiratory apnea, the peak e′ and s′ were recorded throughout the course of five consecutive cardiac cycles, and their averages were then calculated. The average velocities at both the septal and lateral locations were used to calculate E/e′ and E/(e′ × s′). Patients were divided into two groups after being discharged from the hospital according to their E/(e′ × s′) values: Group I had E/(e′ × s′) ≤ 1.6, and Group II had E/(e′ × s′) >1.6. The TDI measurements were taken again 90 ± 10 days later, or around three months after the hospital discharge. E/(e′ × s′) worsening was described as a value higher than the discharge value that had been previously established.

An expert echocardiographer conducted each measurement. We looked at the variabilities between and among observers for E/e’, s’, and E/(e′ × s′). One observer conducted measurements in a group of thirty randomly chosen subjects twice; the other two investigators were in the dark about each other’s measurements and the research time point.

### 2.3. Clinical Variables Recorded

The prognostic model took into account the clinical characteristics that were noted upon hospital discharge, including age, sex, body mass index, heart rate, mean arterial pressures, etiology of heart failure, NYHA functional class, and N-terminal pro-brain natriuretic peptide (NT-proBNP) values. Additionally, the primary treatment classes’ prescriptions for HF were documented.

### 2.4. Clinical Outcome

Both a cardiac mortality and a hospital admission brought on by worsening HF were designated as a major event. Cardiac death included deaths that were directly related to heart conditions, such as congestive heart failure, or unexpected deaths. Electronic medical records and phone conversations with patients or their families were used to gather follow-up data. In this study, we aimed to present the 3-year follow-up outcomes of patients with acute heart failure admitted to the Institute of Cardiovascular Diseases, Timișoara.

### 2.5. Statistical Analysis

Numerical data were presented as the mean ± standard deviation for continuous variables, whereas proportions were represented as categorical variables. Continuous variables were compared through an independent sample *t*-test, while categorical variables were analyzed using the chi-square test. The prognostic factors that were shown to be strongly connected with cardiac events in a univariate analysis were explored using a multivariate approach using a Cox proportional hazard model that used a forward stepwise method. Heart event-free survival rates were computed using Kaplan–Meier analysis, and event rates were compared using log-rank testing. At the time of death, patients who died from non-cardiac reasons were censored, or deemed non-events. E/e′, s′, and E/(e′ × s′) intra-observer and inter-observer variabilities were calculated using the root-mean-square method to calculate the coefficient of variation (CV) and the intraclass correlation coefficient. All statistical tests assumed a significance level of 0.05. Statistical analyses were conducted using SPSS version 26.0 (SPSS Inc., Chicago, IL, USA).

## 3. Results

The current study included 212 consecutive, hospitalized patients, with HF and NYHA class I or II, in sinus rhythm (61 ± 11.4 years; 64 women). The mean LVEF was 46 ± 14.3%. The underlying causes of HF were attributed to coronary artery disease in 148 patients, non-ischemic cardiomyopathy in 34 patients, or systemic hypertension in 30 patients. All patients had recordable mitral annular velocities at both sites. Patient group characteristics are detailed in Table 2. Notably, Group II patients exhibited significantly elevated SPAP and NT-proBNP levels, along with larger LA and LV dimensions. Additionally, they demonstrated higher values for E, the E/A ratio, and the E/e′ ratio, while displaying a lower E-deceleration time, LVEF, e′, and s′. Factors such as age, sex, heart rate, body mass index, NYHA functional class, RV, ROA, and GMI showed no notable differences between the groups.

**Figure 1:**
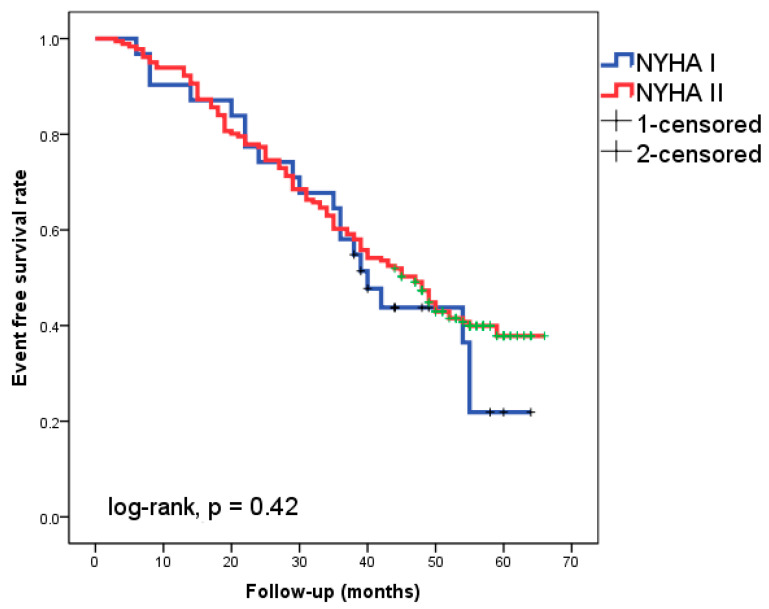
Kaplan–Meier survival curves in the 212 patients with heart failure according to New York Heart Association (NYHA) functional class I or II.

Of the patients followed-up with for 49 ± 11 months, 46.7% experienced cardiac events. Eight patients (3.8%) experienced cardiac death as the initial cardiac episode, while 91 patients (42.9%) experienced HF readmission. The Kaplan–Meier analysis did not show a significantly different event-free survival rate between patients with NYHA class I and II, respectively (Figure 1, log-rank, *p* = 0.42).

**Figure 2:**
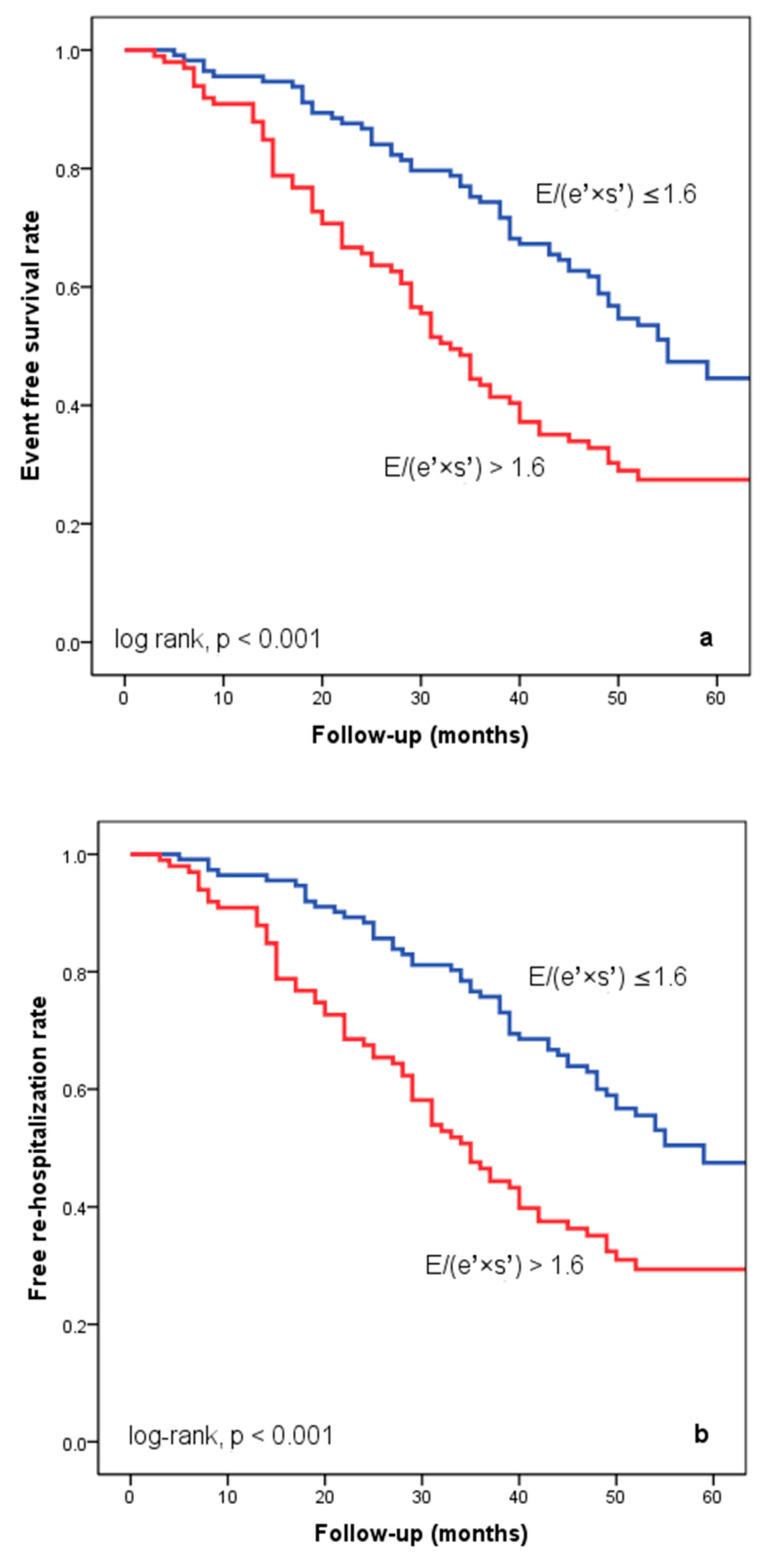

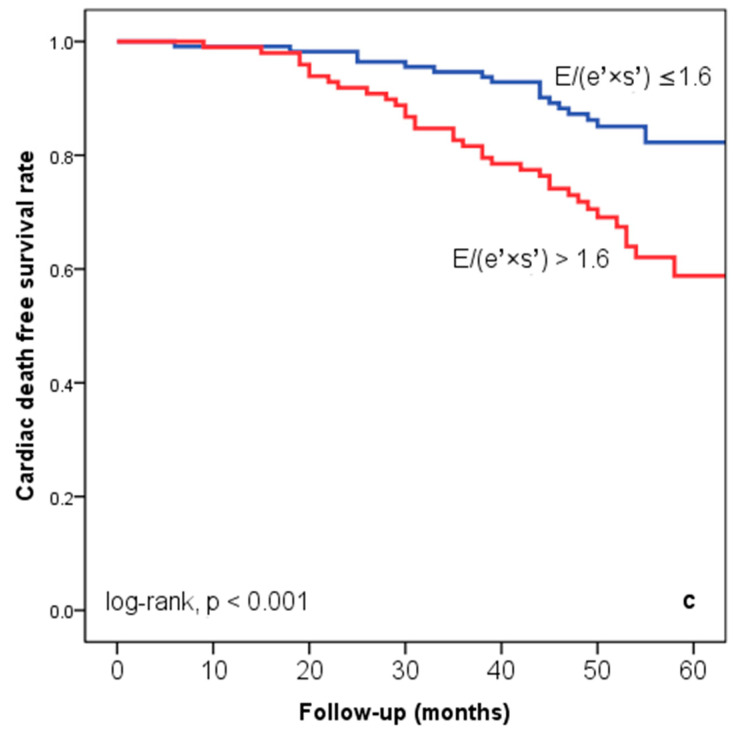
Kaplan–Meier event-free survival curves for cardiac events (**a**), re-hospitalization (**b**), and cardiac death (**c**) in patients at an early stage of heart failure based on the E/(e′ × s′) ratio at hospital discharge, with values either below or above 1.6. E represents early diastolic transmitral velocity, e′ signifies early mitral annular diastolic velocity, and s′ denotes systolic mitral annular velocity.

**Figure 3:**
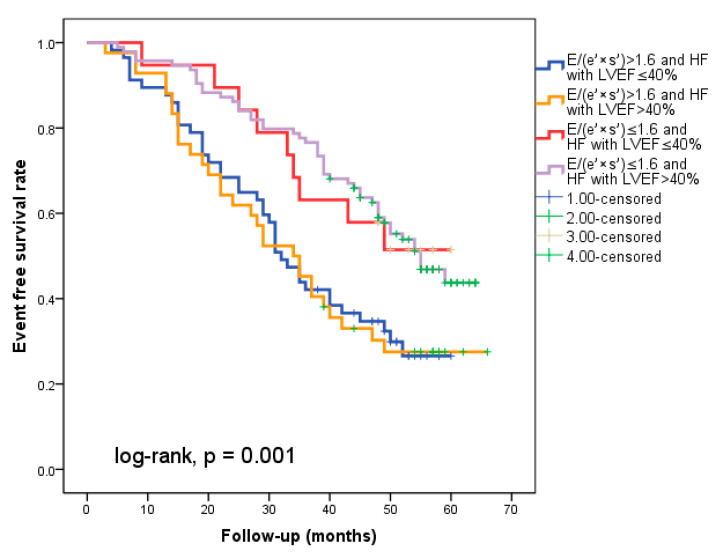
The figure shows that regardless of the type of heart failure (with reduced EF (</=40%) or over 40%), an index over 1.6 indicates a poor prognosis.

At hospital release, Group II’s mean E/(e′ × s′) was 2.8 ± 0.95, whereas Group I’s mean E/(e′ × s′) was 0.91 ± 0.35 (*p* < 0.001). Group II had significantly more occurrences of the composite endpoint (79 events, 70.7% versus 57 events, 50.4%; *p* < 0.001) than Group I. The group exhibiting E/(e′ × s′) > 1.6 (log-rank, *p* < 0.001) had a significantly lower cardiac event-free survival rate over the follow-up period, according to the Kaplan–Meier analysis. (Figure 2a). The same group had higher re-hospitalization and cardiac death rates (Figure 2b,c). The cohort patient group had 76 patients with HF with a reduced EF (LVEF ≤ 40%) (24 patients with functional class NYHA I, and 112, NYHA II) and 136 patients with an LVEF > 40% (7 patients with NYHA I, and 69, NYHA II) (*p* = 0.108). Irrespective of the LVEF value, an index > 1.6 was associated with an unfavorable prognosis (Figure 3).

The variables that showed predictive value for cardiac events (*p* < 0.05) in univariate Cox regression analysis are listed in Table 3: LA volume, SPAP, E, E/A ratio, E/e′, s′, and E/(e′ × s′) are among the NT-proBNP levels. On the other hand, the univariate analysis revealed that cardiac events were not significantly correlated with age, sex, heart rate, blood pressure, NYHA class, aetiology of HF (coronary artery disease, etc.), treatment, indexed LA volume, LVEF, LV end-diastolic volume index, LV end-systolic volume index, E-deceleration time, A, e′, RV, or ROA. Subsequently, a multivariate analysis was conducted using univariate important predictors to track the occurrence of cardiac events. The best independent predictor at 36 months of follow-up was found to be the E/(e′ × s′) prior to discharge (HR = 1.55, 95%, CI = 1.100–1.202, *p* = 0.012).

**Figure 4:**
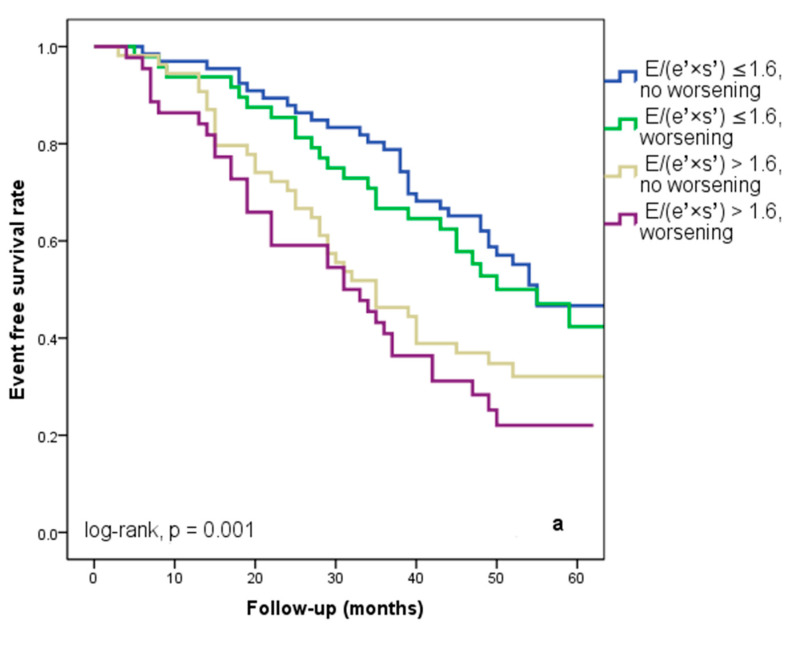

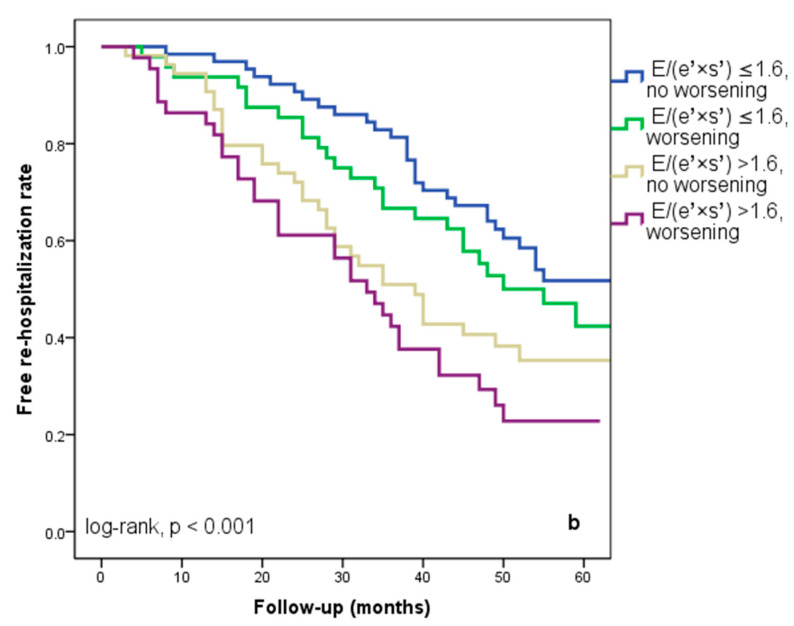

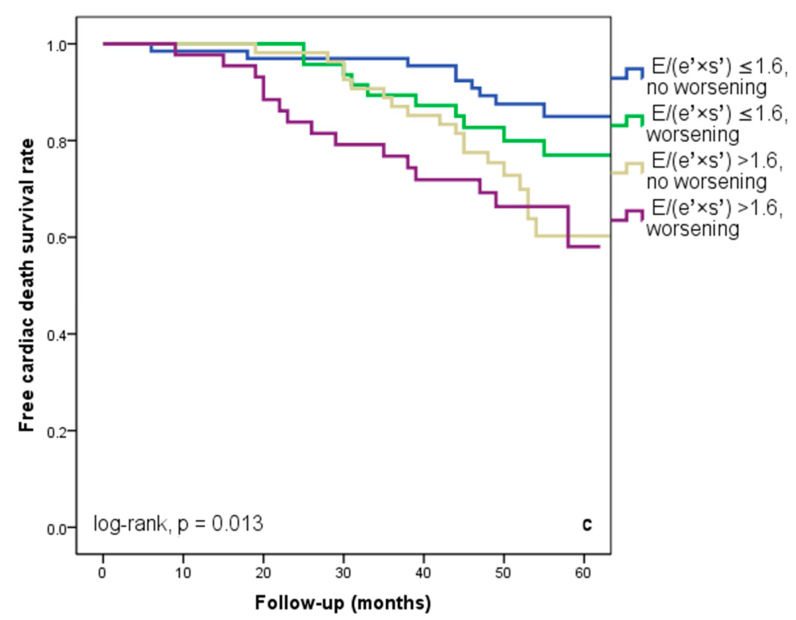
Kaplan–Meier event-free survival curves for cardiac events (**a**), re-hospitalization (**b**), and cardiac death (**c**) in patients at an early stage of heart failure according to the initial E/(e′ × s′) ratio at hospital discharge (above or below 1.6) and worsening or no worsening after three months. E represents early diastolic transmitral velocity, e’ signifies early mitral annular diastolic velocity, and s’ denotes systolic mitral annular velocity.

We found that 92 patients (43.3%) worsened in their E/(e′ × s′) ratio three months after being discharged from the hospital. Out of these individuals, 44 (20.7%) had an initial E/(e′ × s′) value that was higher than 1.6. Regardless of the E/(e′ × s′) value at study inclusion, as Figure 3a illustrates, E/(e′ × s′) worsening was linked to a lower cardiac event-free survival rate (25% versus 33.3% in patients with the initial E/(e′ × s′) > 1.6 and 47% versus 50% in those with E/(e′ × s′) ≤ 1.6 at hospital discharge, log-rank, *p* = 0.001). The patients who initially had an E/(e′ × s′) ratio greater than 1.6 and whose ratio continued to worsen after three months were the subgroup with the worst prognosis for cardiac events, HF rehospitalizations, and cardiac fatalities (Figure 4a–c). In total, 52 patients, or 24.5% of the sample, experienced cardiac-related deaths throughout the course of the follow-up period. Group I and Group II had comparable rates of non-cardiac death [4 (3.5%) vs. 3 (3.03%), *p* = 0.72].

The E/e′, s′, and E/(e′ × s′) had intra-observer intraclass coefficients of 0.94 (CV 2.7%), 0.95 (CV 3%), and 0.93 (CV 3.2%), in that order. Similarly, for the E/e′, s′, and E/(e′ × s′), the inter-observer intraclass coefficients were 0.91 (CV 2.9%), 0.92 (CV 3.2%), and 0.90 (CV 3.1%), correspondingly.

## 4. Discussion

In the present study, we analyzed the impact of the E/(e′ × s′) index to predict cardiac events, the worsening of HF, and cardiac death in HF patients with NYHA class I or II, in sinus rhythm. An E/(e′ × s′) ratio >1.6 emerged as a robust predictor of forthcoming cardiac events, surpassing various other echocardiographic parameters: NYHA functional class, coronary artery disease, and NT-proBNP levels. Its predictive power notably intensifies when linked with deterioration three months later.

Cardio-vascular imaging, especially echocardiography, is essential for the diagnosis and treatment of heart failure [2]. Because early treatment can positively influence this progression, the early diagnosis of LV dysfunction is essential. Previous studies have demonstrated the significant prognostic significance of the LVEF, LA size, and LV volume indices for outcomes in patients with heart failure [17,18,19]. However, after conducting multivariate analyses, variables that had been initially found in univariate analysis as outcome predictors—like the SPAP, LA volume, E, and E/A ratio—were eliminated from the study. The better performances of the TDI parameters may be partially explained by the dependence of mitral flow on variables such as age, LA pressure, volemic state, and myocardial relaxation [4].

The use of TDI, which is compatible with echocardiography equipment from many manufacturers, reduces the demand to trace endocardial contours, which is necessary for LV volumes and LVEF assessment [17]. More precise and sophisticated measurements are required in clinical trials and to guide treatment for individual patients. Several studies have demonstrated that the e′ velocity and E/e′ ratio are highly predictive of adverse events following acute myocardial infarction, and in patients with LV dysfunction, it was recently demonstrated that abnormal LV diastolic function is missed by using the reductive index E/e′ alone [4,8,20,21,22,23]. Furthermore, Sharifov et al. recently showed that there is insufficient data to substantiate the claim that E/e′ can accurately assess the LV filling pressure in patients with maintained LVEFs, which is commonly found in patients classified as NYHA class I or II [24]. This may be explained in part by the similar preload sensitivity of E and e’ so that their ratio does not change [8].

One of the main causes of the development of HF is diastolic impairment combined with systolic dysfunction. The LV long-axis function analysis provided insightful supplementary data for the noninvasive evaluation of the HF prognosis. However, the TDI enables a modern assessment of the s′ wave, or longitudinal systolic function, which is impacted differently and at distinct phases in HF [8,25]. As these interact with the prognosis, Biering-Sørensen et al. recommended evaluating TDI velocities (e′ and s′) jointly [26]. Independent of traditional echocardiographic criteria, a pattern of low systolic and diastolic performances as determined by the TDI is a critical sign of an unfavorable prognosis. Our research showed that the E/(e′ × s′) ratio—which combines an LV systolic performance marker (s′) with an HF diastolic indicator (E/e′)—proved to be a more useful metric for prognostic evaluation in an unselected HF sample [10]. Hirata et al. observed that patients with higher-risk heart failure who were at risk of readmission and cardiac death could be identified using a composite index that included an LVEF ≤ 40% and E/e′ratio > 15 [27]. Given the reduced ability of s′ to detect LV dysfunction in people with a maintained LVEF, the E/(e′ × s′) index emerges as a more robust metric [13].

Participants with NYHA I and II were matched to the same number of participants with NYHA III and IV in a sub-analysis of the Digitalis Investigational Group study. The results show that the death rates were 34% against 42%, and the all-cause hospitalization rates were 66% compared to 71%, respectively [7]. Additionally, this highlights the notably poor results for those who are purportedly less sick, suggesting that all HF patients require an equally rigorous course of treatment. There is still a comparatively high burden of morbidity and mortality among patients with NYHA class I and II. In practical practice, prognosis prediction helps identify the subset of high-risk patients who could benefit from more intensive heart failure treatment [28]. Early treatment can have a positive impact on the progression of HF. After comparing the E/(e′ × s′) index to numerous other clinical and paraclinical characteristics, especially when linked to its worsening three months later, we found that it was the strongest predictor of cardiac events, the worsening of HF, and cardiac death in NYHA class I or II.

The study group exhibited a high prevalence of coronary artery disease, raising the possibility of ischemic events contributing to the observed cardiac events [2]. Severe myocardial ischemia cause complex changes defined as LV remodeling that affect the ventricular functions and prognosis [22,29]. Biering-Sørensen et al. investigated the color Doppler parameters in patients treated with primary coronary angioplasty and found that the s’ and e’ values predict adverse cardiac events. Another small study on people with ischemic HF showed that 19% and 60% of patients with NYHA I or II, respectively, had dysfunctional but viable myocardium [26,30]. Within our study, the presence of coronary artery disease did not emerge as a predictor for cardiac events.

The severity of stable chronic heart failure as indicated by the NYHA functional classification is reflected in the NT-proBNP levels. A favorable prognosis in the medium term is predicted by high levels of NT-proBNP, while low levels are linked to an excellent prognosis [2,9]. After receiving the necessary medical care, we carried out NT-proBNP determination and echocardiography. Our data’s statistical analysis confirms that while NT-proBNP has predictive value, it is not as good as E/(e′ × s′). In a similar vein, Lim et al. showed that an aberrant echocardiography was more sensitive than plasmatic NT-proBNP levels at predicting outcomes [31].

A few limitations should be taken into account when interpreting our results. Despite the relatively small sample size in this study, we were nonetheless able to make several important findings. One drawback of the NYHA categorization, which is currently based on a physician’s experience, is its lack of consistency and accuracy. Previous studies [1] have revealed low concordance (only 54–56%) between two cardiologists when asked to characterize the NYHA class of patients with mild to moderate symptoms. One may argue that the study’s use of standard echocardiographic assessments rather than more advanced techniques (such as strain imaging) is both a drawback and a strength. The drawback is that sub-clinical impairments of both systolic and diastolic function have been found to be more sensitively detected via strain imaging. Our parameter’s strength lies in its simplicity of use, which is a result of its inclusion in the majority of contemporary echo machines. This allows it to be easily applied for patient bedside assessments. We measured only the TDI at the medial and lateral mitral annulus, and we neglected to look at the anterior and posterior velocities, which could have revealed more details. The study center functioned as a tertiary invasive center, and therefore, the study population may not reflect a general population of patients with HF.

## 5. Conclusions

According to our findings, the TDI-derived index E/(e′ × s′) among HF patients in NYHA class I or II in sinus rhythm is a substantial independent long-term predictor of cardiac death or HF-related hospitalization. When paired with its worsening three months later, an E/(e′ × s′) ratio > 1.6 can specifically identify high-risk patients who are susceptible to cardiac events. It is crucial to remember that our study was limited to one center; therefore, it would be more credible if it were validated at other centers or through multicenter trials.

## Figures and Tables

**Table 1:** Inclusion and exclusion criteria in the patient group.

Inclusion Criteria	Exclusion Criteria
Heart failure, in sinus rhythm, hospitalized with NYHA functional class I or II, with or without RV disfunction.	Symptoms or clinical and instrumental signs of acute coronary syndrome or acute myocarditis at the first medical contact (patients hospitalized with heart failure after 4 weeks were enrolled in the study); Inadequate echocardiographic images; Coronary revascularization during follow-up; Significant primary valvular heart disease (except mild forms not hemodynamically relevant); Cardiac pacemaker/defibrillator; Congenital heart disease; Renal failure (serum creatinine >1.3 mg/dL); Malignant neoplasia; Anemia (hemoglobin <12 mg/dL in women and <13 mg/dL in men); Endocrine disorders (hypo- and hyperthyroidism, hyperaldosteronism); Pericardial disease; Primary pulmonary hypertension; Ascendent or descendent aortic diseases.

**Table 2:** Baseline characteristics of the study groups. A = late transmitral flow velocity; ACEI = angiotensin-converting enzyme inhibitor; E = early diastolic transmitral flow velocity; e′ = early mitral annular diastolic velocity; LV = left ventricle; NT-proBNP = N-terminal pro-brain natriuretic peptide; s′ = systolic velocity of mitral annulus.

Characteristics	Group I (*n* = 113)	Group II (*n* = 99)	*p*-Value
**Clinical characteristics**			
Age, years	61 ± 12	62 ± 11	0.79
Sex, female/male	35/78	29/70	0.88
Body mass index, kg/m^2^	27.2 ± 4.51	26.9 ± 4.71	0.81
Heart rate, beats/min	74 ± 20	78 ± 21	0.59
Mean arterial pressure, mmHg	99.8 ± 14.8	96.8 ± 17.1	0.52
Coronary artery disease, *n* (%)	84 (39.7)	64 (30.2)	0.13
Non-ischemic cardiomyopathy, *n* (%)	18 (8.5)	16 (7.5)	0.31
Systemic hypertension, *n* (%)	17 (8)	13 (6.1)	0.22
NYHA class, *n*	16 (I)/97 (II)	15 (I)/84 (II)	0.85
NT-proBNP, pg/mL	600 ± 921	1278 ± 2372	0.005
**Therapy in admission**			
Beta blocker, *n* (%)	110 (97.3)	82 (82.8)	0.001
ACEI/angiotensin receptor antagonist, *n* (%)	112 (99.1)	93 (93.9)	0.06
Diuretics, *n* (%)	82 (72.5)	70 (70.7)	0.87
Digoxin, *n* (%)	11 (0.9)	13 (1.3)	0.37
Nitrates, *n* (%)	77 (68.1)	66 (66.6)	0.88
**Echocardiographic indices**			
LV end-diastolic volume index, mL/m^2^	91 ± 31	109 ± 35	0.011
LV end-systolic volume index, mL/m^2^	44 ± 28	65 ± 22	0.009
LV ejection fraction, %	51 ± 12	40 ± 12	0.001
Left atrial volume, mL	71 ± 23	103 ± 39	0.001
Indexed left atrial volume, mL/m^2^	39 ± 13	55 ± 21	0.001
Systolic pulmonary artery pressure, mmHg	36 ± 8.5	43 ± 14	0.001
Mitral regurgitant orifice area, mm^2^	24 ± 7.7	25 ± 9.8	0.51
Mitral regurgitant volume, mL	36 ± 14.8	38 ± 13.9	0.75
E, cm/s	67 ± 22	88 ± 25	0.001
E/A ratio	0.84 ± 0.35	1.52 ± 0.93	0.001
E-deceleration time, ms	176 ± 65	162 ± 76	0.16
Global myocardial index	0.86 ± 1.23	0.65 ± 1.1	0.21
e′, cm/s	9.5 ± 3.6	6.5 ± 2.1	0.001
E/e′ ratio	7.5 ± 2.3	14.2 ± 4.1	0.001
s′, cm/s	8.9 ± 2.3	5.3 ± 1.1	0.001
E/(e′ × s′) ratio	0.91 ± 0.35	2.8 ± 0.95	0.001

**Table 3:** Clinical, laboratory, and echocardiographic variables at hospital discharge associated with cardiac events in the Cox univariate and multivariate analysis. A = late diastolic transmitral velocity; CI = confidence interval; E = early diastolic transmitral velocity; e′ = mitral annular diastolic velocity; HR = hazard ratio; LVEF = left ventricular ejection fraction; s′ = systolic velocity of mitral annulus; NA = not applicable; NT-proBNP = N-terminal pro-brain natriuretic peptide; SPAP = systolic pulmonary artery pressure.

Variables	Univariate HR (CI 95%)	*p*-Value	Multivariate HR (CI 95%)	*p*-Value
NT-proBNP level	1.32 (1.151–1.511)	0.001	1.103 (0.746–1.626)	0.531
LA volume	1.005 (1.001–1.009)	0.012	1.005 (0.995–1.006)	0.750
SPAP	1.019 (1.006–1.033)	0.005	1.008 (0.992–1.025)	0.347
E velocity	1.009 (1.002–1.015)	0.008	1.005 (0.993–1.016)	0.436
E/A ratio	1.313 (1.062–1.623)	0.012	0.941 (0.670–1.322)	0.725
E/e′ ratio	1.051 (1.016–1.088)	0.004	0.929 (0.851–1.015)	0.103
s′ velocity	0.875 (0.814–0.940)	0.001	1.023 (1.003–1.043)	0.022
E/(e′ × s′) ratio	1.344 (1.172–1.540)	0.001	1.556 (1.100–2.202)	0.012

## Data Availability

The data presented in this study are available on request from the corresponding author.
